# What are the prospects for citizen science in agriculture? Evidence from three continents on motivation and mobile telephone use of resource-poor farmers

**DOI:** 10.1371/journal.pone.0175700

**Published:** 2017-05-04

**Authors:** Eskender Beza, Jonathan Steinke, Jacob van Etten, Pytrik Reidsma, Carlo Fadda, Sarika Mittra, Prem Mathur, Lammert Kooistra

**Affiliations:** 1 Laboratory of Geo-Information Science and Remote Sensing, Wageningen University, Droevendaalsesteeg 3, PB Wageningen, The Netherlands; 2 Plant Production Systems, Wageningen University, AK Wageningen, The Netherlands; 3 Humboldt-Universität, Berlin, Germany; 4 Bioversity International, Turrialba, Costa Rica; 5 Bioversity International, Addis Ababa, Ethiopia; 6 Department of Forest and Wildlife Ecology, University of Wisconsin-Madison, 1630 Linden Drive, Madison, WI, United States; 7 Bioversity International, NASC Complex, Pusa Campus, New Delhi, India; Cornell University College of Agriculture and Life Sciences, UNITED STATES

## Abstract

As the sustainability of agricultural citizen science projects depends on volunteer farmers who contribute their time, energy and skills, understanding their motivation is important to attract and retain participants in citizen science projects. The objectives of this study were to assess 1) farmers’ motivations to participate as citizen scientists and 2) farmers’ mobile telephone usage. Building on motivational factors identified from previous citizen science studies, a questionnaire based methodology was developed which allowed the analysis of motivational factors and their relation to farmers’ characteristics. The questionnaire was applied in three communities of farmers, in countries from different continents, participating as citizen scientists. We used statistical tests to compare motivational factors within and among the three countries. In addition, the relations between motivational factors and farmers characteristics were assessed. Lastly, Principal Component Analysis (PCA) was used to group farmers based on their motivations. Although there was an overlap between the types of motivations, for Indian farmers a collectivistic type of motivation (i.e., contribute to scientific research) was more important than egoistic and altruistic motivations. For Ethiopian and Honduran farmers an egoistic intrinsic type of motivation (i.e., interest in sharing information) was most important. While fun has appeared to be an important egoistic intrinsic factor to participate in other citizen science projects, the smallholder farmers involved in this research valued ‘passing free time’ the lowest. Two major groups of farmers were distinguished: one motivated by sharing information (egoistic intrinsic), helping (altruism) and contribute to scientific research (collectivistic) and one motivated by egoistic extrinsic factors (expectation, expert interaction and community interaction). Country and education level were the two most important farmers’ characteristics that explain around 20% of the variation in farmers motivations. For educated farmers, contributing to scientific research was a more important motivation to participate as citizen scientists compared to less educated farmers. We conclude that motivations to participate in citizen science are different for smallholders in agriculture compared to other sectors. Citizen science does have high potential, but easy to use mechanisms are needed. Moreover, gamification may increase the egoistic intrinsic motivation of farmers.

## 1. Introduction

Public participation has a long and distinguished tradition in agricultural research. Over the last decades, numerous methodologies have been developed to address the participation of farmers as agricultural end-users in trial design, innovation development, different steps of plant breeding, and other fields of research [1–3]. Important objectives of involving farmers in research include creating synergies between local and formal innovation, and increasing the practical impact of research [4, 5]. Participatory methodologies in the agricultural sciences usually involve limited numbers of farmers, often trained by researchers and living in close proximity to the research facility, and scaling is usually difficult due to requirements in training and farmer group organization [6]. Yet, given the strong heterogeneity of socio-economic requirements and environmental conditions in many locations, there is increasing interest in methodologies that facilitate the engagement of larger numbers of farming households and environments.

In the last fifteen years, modern communication tools have enabled the emergence of participatory research methodologies involving very high numbers of contributors via crowdsourcing [7, 8]. Although the term may include any participatory research, such methodologies are usually referred to as ‘citizen science’, and have now become widely established and led to many peer-reviewed publications in the ecological and biological disciplines [9–11]. In citizen science projects, a large number of volunteers individually participate in crucial activities of formal research. These projects have accomplished tasks that traditional research often cannot, due to restricted resources. The accumulated time dedicated to the crowdsourced research task, the number of contributions, and, in many cases, the geographic spread of data entries often exceed the capacities of traditional research. Successful examples include national surveys of bird migration [12], or citizens classifying the water quality of nearby water bodies [13]. Only now, similar research methodologies are under development for the agricultural sciences, offering new opportunities for the scaling and specification to local context of agricultural research.

Although other factors also play a role, recent literature suggests two important preconditions for establishing successful crowdsourced research. Firstly, since the remote network of participants is a key characteristic of crowdsourced research, participants must have access to digital communication infrastructure. And secondly, since participation is voluntary, participants need to be motivated [14]. In Self-Determination Theory (SDT) two basic types of motivations are distinguished: intrinsic motivation, which refers to “doing something because it is inherently interesting or enjoyable”, and extrinsic motivation, which refers to “doing something because it leads to a separable outcome” [15]. Research about volunteers’ motivation to participate in citizen science has suggested a key role of egoistic affective goals, like fun and the experience of participating in a meaningful activity [16, 17]. So, to address affective parts of motivation in designing for large-scale participation, many recent citizen science projects have introduced elements of *gamification* [7]. Gamification refers to the application of design elements from games to a non-game context, with the end goal of improving the user experience, and, eventually, motivating participation [18]. Empirical research demonstrates that gamification can encourage some people to use an application more often [19] and to derive greater enjoyment from their use of an application [20, 21].

One simple way to gamify citizen science is to provide extrinsic incentives such as score boards, badges or progressive ranks. But many projects rely on more sophisticated motivational design. For example, the citizen science project eBird provides game-type incentives like personal bird lists, user rankings or rare bird alerts, the introduction of which contributed to a strong increase in participant numbers [22]. These incentives draw on intrinsic motivation rather than the extrinsic motivation of scoreboards and social rewards. Many successful citizen science projects rely on intrinsic motivation, like participants’ interest in learning, developing skills, and social exchange [23–25]. We are specifically interested in the relation between intrinsic and extrinsic motivation in farmer citizen science.

The goal of our research is to contribute to the design and development of citizen science methodologies for the agricultural sciences that can effectively engage high numbers of smallholder farmers in developing countries. According to[26], digital citizen science is founded on two facilitating pillars: motivational and technological. Our specific objectives are therefore to assess 1) farmers’ motivations to participate as citizen scientists and 2) farmers’ mobile telephone usage. Although the mantra “easy, fun and social” [7] points the way, more context-specific analysis is needed. As citizen science methodologies for the agricultural sciences are just emerging, it is questionable to what extent insights from motivational studies with participants in citizen science projects from other disciplines may be generalized. Given the strong link of the research topic to their families’ livelihoods, farmers’ motivation may differ substantially from the motivation revealed in previous studies, where participants usually engaged as a leisure-time activity. Thus, to be able to design methodologies for large-scale agricultural citizen science, it is crucial to understand what motivates participants.

We draw insights from citizen science projects of crop variety trials which implement ‘triadic comparison of technologies’ (tricot). In this project, farmers participate by planting three varieties of one crop on their own farms, and reporting simple observations to an implementing body, like an NGO or a research organization [27, 28]. Data and information exchange is already facilitated by mobile phone technology, which we see as a simple technological interface that allows observing opportunities and constraints of future digitalized citizen science trials in practice.

In order to derive conclusions that may contribute to design principles for agricultural citizen science, we were interested in what are the roles of different drivers of motivation for the engagement of participants. We therefore assessed the relative importance of egoistic, collectivistic, and altruistic motives. Motivation may not be homogeneous among participants: individual differences in, for example, age, gender, education level or country may influence what motivates participants (and what does not). When different groups of participants can be distinguished by their motivation, citizen science projects may be specifically designed to offer different roles for participants, with different types of participation. Hence, we tested for interactions between motivation and farmers’ characteristics. We addressed the following research questions: What motivates farmers to participate in agricultural citizen science? Which different groups of participants can be distinguished with regard to their motivation?

To be able to make statements about potential future use of communication technology in citizen science, it is vital to understand opportunities and constraints related to the use of mobile phone technology by current participants in citizen science, who represent an already-motivated sub-sample. We studied the habits in usage of mobile phones, as well as the availability and distribution of related resources like literacy and airtime credit among participants in tricot. Here, too, it is interesting to identify discrete groups of participants, since different roles and different types of participation in the citizen science project may also be offered in order to address the variety of technology user profiles. With our research, we want to answer the following questions: In what ways are mobile phones used by participants in citizen science? Which opportunities and constraints does this experience bring along? We then conclude by analyzing how these findings may translate into design principles for agricultural citizen science projects.

## 2. Material and methods

### 2.1. Seeds for Needs initiative

**Fig 1:**
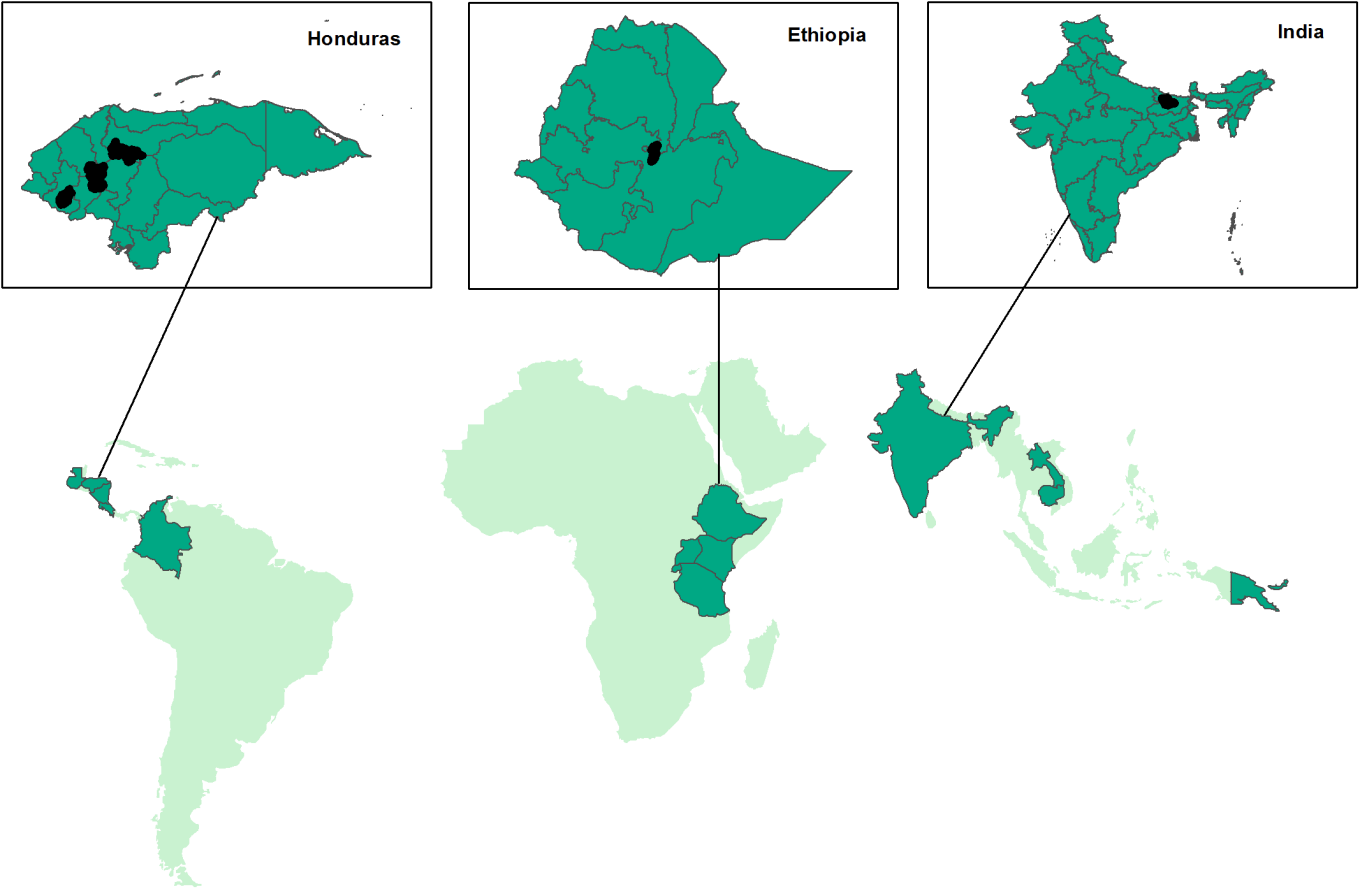
Overview of countries where the Seeds for Needs initiative is running (lower map) and locations in Honduras, Ethiopia and India (upper insets) where the surveys were conducted (black polygons inside the three countries).

Seeds for Needs is a Bioversity International initiative involving more than 20,000 smallholder farmers in 14 countries (Fig 1), to explore how agricultural biodiversity can minimize the risks associated with climate variability (www.bioversityinternational.org/seeds-for-needs/). The main idea of the initiative is: if farmers have the opportunity to access better information and different varieties, they are better able to choose what is appropriate for their conditions and cope with unpredictable weather [27, 28]. The Seeds for Needs initiative addresses the issue of access to information and seed variety by exposing farmers to more crop varieties and increasing their knowledge about different traits. Since 2011, the initiative has been using a crowdsourcing approach called triadic comparisons of technologies (tricot): each farmer receives three randomly-assigned varieties from a larger pool of varieties, to compare with their own varieties. By carrying out these on-farm mini-trials with a small number of varieties, many farmers can participate voluntarily as citizen scientists. The initiative involves farmers directly in evaluating and selecting varieties, and provides valuable feedback about preferred traits to researchers. Mobile phones are also used by the initiative to communicate with farmers. Weather sensors, known as iButtons, have been setup in farmers’ fields to record local temperature and humidity [29]. The collected data is compared with feedback from farmers on crop performance. The ClimMob data analysis software has been developed to help identify trends and give farmers feedback based on the collected data [28]. The participation of farmers is voluntary.

### 2.2 Study areas

We chose India, Ethiopia and Honduras to explore the motivation of the farmers to participate in crop improvement trials using the crowdsourcing approach as citizen scientists. The main reason why we focused on these three countries is because of their geographical locations (in three continents) and duration of the Seeds for Needs initiative. The initiative has been testing the crowdsourcing for crop improvement trial approach in these three countries at least since 2013.

#### 2.2.1 India

The Seeds for Needs initiative started with 10 farmers in 2010. The crowdsourcing approach was first implemented in 2012 and, within three years, included 15,000 rice and wheat growing farmers in 24 districts in four states [29]. For the current research, data were collected from 300 farmers in 30 villages from Muzaffarpur (26.17° N, 85.42° E), Samastipur (25.86° N, 85.78° E) and Vaishali (25.99° N, 85.13° E) districts in the state of Bihar.

#### 2.2.2 Ethiopia

The Seeds for Needs initiative first started in Ethiopia in 2009. From 25,000 accessions of durum wheat and barley, 500 were short listed using Geographic Information System (GIS) technology and characterization. Out of this short list, farmers and scientists selected 50 to test for local adaptation. The crowdsourcing approach was first tested in two areas involving 200 farmers in 12 villages covering 350 km^2^ with different climatic conditions. Currently the project is working with more than 1500 farmers. For our current research, data were collected from 94 farmers in 9 Kebele’s (smallest administrative unit) from Gimbichu (8.83° N, 39.17° E) and Lume districts (8.58° N, 39.17° E) of the Oromia Region.

#### 2.2.3 Honduras

The Bean Research Program at *Zamorano*, a private agricultural school, has collaborated with two local NGOs in the diffusion and evaluation of 18 traditional and improved varieties of common bean within Seeds for Needs, from 2013 to 2015. Around 200 farming households in four different regions participated in the project. For this research, data were collected from 32 farmers in 9 municipalities from Comayagua (14.46° N, 87.65° W), Intibucá (14.32° N, 88.15° W), Lempira (14.58° N, 88.58° W), Santa Barbará (14.91° N, 88.23° W) and Yoro (15.13° N, 87.1° W) departments.

### 2.3 Theoretical framework

Motivation is a concept used in behavioral science to explain the “initiation, direction, intensity, persistence, and quality” of behavior [30]. We follow the definition by Brophy [30], where motives are “hypothetical constructs used to explain why people are doing what they are doing”. Participation in research is necessarily participation in collective action, and Batson et al. [31] proposed four types of motives for participation in activities with collective goals: *egoism* (the ultimate goal of involvement is increasing one’s own welfare), *altruism* (increasing other persons’ welfare), *collectivism* (increasing the welfare of a group one belongs to), and *principlism* (to uphold one or more moral principles). In this study, we assess the importance of these types of motives for farmers’ engagement in a citizen science project.

We distinguish egoistic motivation driven by intrinsic motives, which involves inherent satisfaction, and extrinsic motives, which are believed to lead to some desirable, separable outcome [15]. This study applies incentive theory to study motivation, seeing human beings as fundamentally active, proactively pursuing goals, and responsive in their behavior to external reinforcement, i.e., incentives [32]. Therefore, we seek to identify the most important incentives that researchers may set to increase farmer participation in citizen science.

### 2.4 Survey design: Selection of motivational factors

As starting point for this study, a literature study about the motivation of people to participate in different crowdsourcing and citizen science activities was conducted. Motivational factors identified by previous studies were used as a starting point to develop a questionnaire for semi-structured interviews (Table 1).

**Table 1:** Motivational factors identified by previous studies and used in this study. Typology is based on the framework of Batson et al. [31].

No.	Motivational factors	Code	*Type(s) of motivation*	References
Mot 1	Want to contribute to scientific research	Contributing	Collectivistic	[16, 23, 24, 33, 34]
Mot 2	Wish to pass free time (fun)	Pastime	Egoistic (Intrinsic)	[16, 23, 35, 36]
Mot 3	Interest in sharing information	Sharing Info.	Egoistic (Intrinsic)	[24]
Mot 4	Expectation of something in return	Expectation	Egoistic (Extrinsic)	[34, 37]
Mot 5	Interest in networking with experts	Expert Interaction	Egoistic (Extrinsic)	[25]
Mot 6	Interest in networking with other community members	Community Interaction	Egoistic (Extrinsic)	[16, 35–39]
Mot 7	Wish to help the researcher(s)	Helping	Altruistic	[16, 23, 25, 31, 39]

We interviewed farmers about their motivation to participate in the citizen science project using a semi-structured interview format. We asked farmers whether they were interested in continuing participation in the future, and to elaborate their answer. Then, we presented the seven potential motivational factors for participation (Table 1), including intrinsic and extrinsic egoistic, as well as collectivistic and altruistic motivational factors, and asked for the level of importance of each motivational factor for their personal motivation to participate in crop variety trials.

In addition, three open questions about motivation were included in the survey. Firstly, respondents were given the opportunity to express additional motivational factors that were not included in the options. Secondly, farmers who had ranked motivational factor 4 (I participate because I expect something in return from the expert) “Important” or “Very important” were asked to specify the incentives they expected from the expert or from the citizen science process.

Lastly, farmers were asked whether they would expect a reward (Yes/No) for sharing farm information in the future. Respondents answering “Yes” were asked to specify the type of preferred reward they would like to receive. The latter question was asked only in India and Ethiopia.

### 2.5 Data collection

In 2014 and 2015, we conducted 426 face-to-face structured interviews in three countries; India (300), Ethiopia (94), and Honduras (32). In India, farmers who had participated in more than two growing seasons were selected and data collection was carried out by junior agronomists working for Bioversity International-India. In Ethiopia, researchers and agricultural extension workers of the Ethiopian Biodiversity Institute selected the farmers for interview, giving preference to individuals who had participated in the trials for more than one growing season. The first author conducted the interviews together with project team members of the Ethiopian Biodiversity Institute. In Honduras, farmers were selected by local NGO extension workers, and the second author carried out the interviews. The selection of participants was determined by ongoing activities of the local NGO and no explicit criteria were used to select farmers.

During the structured interview, motivational factors (Table 1) were read to each farmer one by one in their local language and each farmer was asked to mark if they apply to his/her personal motivations for participation in the crowdsourcing for crop improvement trials. Farmers were asked to rank the motivational factors using Likert scales with the values 1 (“Not important at all”), 2 (“Not important”), 3 (“Neutral”), 4 (“Important”) and 5 (“Very important”). Farmers’ characteristics (age, education level, head of household (Yes/No) and gender) and use of mobile phones were also collected during the interview (see S1 Appendix for a complete list of questions).

### 2.6 Data analysis

#### 2.6.1 Quantitative data analysis

Data were first analyzed by frequencies and percentages. Comparisons of motivational factors within each of the three countries were performed using Friedman’s test, a non-parametric model used to test for differences between groups across multiple conditions. This was followed by post-hoc pairwise comparisons using the Wilcoxon signed-rank test. Comparisons of motivational factors between the three countries were performed using the Kruskal-Wallis test followed by post-hoc pairwise comparisons using the Dunn-Bonferroni approach. The Kruskal-Wallis test is a non-parametric statistical test that assesses differences among three or more independently sampled groups on a single, non-normally distributed continuous variable [40]. The test can deal with non-normally distributed data (e.g., ordinal or rank data) [41]. For both tests, the level of significance was set at 0.05 and Bonferroni adjustment was used to account for multiple comparisons [42].

Principal Component Analysis (PCA) was used to group the motivational factors into smaller sets of groups and also to assess the correlation between the motivational factors. We determined how different types of motivation can be explained with farmers’ characteristics (gender, age, education level, household head (Y/N) and country) using Generalized Linear Models (GLM). To create the GLMs, we chose the linear model type, included only main effects, and selected Type III analyses, Wald statistics, and a significance level of *p* = 0.05 for identifying significant relations.

In addition, Redundancy analysis (RDA) was used to identify the most important farmers’ characteristics that explain the variation of farmers’ motivations in the three countries. RDA can assess how much of the variation in the motivational factors values can be explained by the farmers’ characteristics. The suitability of RDA was first identified by a detrended correspondence analysis (DCA) to obtain the gradient length of response variables [43]. The linear ordination method (RDA) was suggested because of the small gradient length (0.8 SD). By using the manual forward selection procedure of Canoco advisor (an expert system built into Canoco 5) [43], the statistical significance of each of the farmers’ characteristics included in the model was calculated by performing Monte Carlo permutation tests (499 unrestricted permutations), testing against the null hypothesis that the factor does not add to the explanation of the motivation data. In this stepwise selection, we chose factors with a threshold of *p* < 0.05 for retention in the model. Moreover, the score scaling type was set to focus on response variable correlations, and response variable scores were divided by standard deviations [43].

The relative relationship between motivational factors (response variables) and farmers’ characteristics (explanatory variables) were demonstrated using triplot diagrams. In the RDA triplot, the correlation between motivational factors and farmers’ characteristics is given by the cosine of the angle between the two vectors [43]. Vectors crossing at right angles indicate a near zero correlation, vectors pointing in the same direction indicate a positive correlation, while vectors pointing in opposite direction show a high negative correlation.

For the last three analyses (i.e., for GLM, PCA and RDA) normalized Likert scale data were used. Missing values were treated as missing listwise in the calculations. All non-parametric tests and GLMs were performed using SPSS version 22 and multivariate analyses (PCA and RDA) were performed using Canoco 5.

#### 2.6.2 Qualitative data analysis

To identify the main motives, effective incentives and farmers’ expectations from experts, the responses from the open-ended survey questions were subjected to a qualitative content analysis method. The open-ended survey questions about farmers’ additional motivations, expectations and types of rewards farmers would like to receive for sharing agronomic information were analyzed qualitatively. This analytical approach involves a close examination of textual data, which is explored inductively for emerging themes relating to the same central meaning [44]. These themes were grouped into coding units, counted and presented graphically. Responses from open-ended questions were analyzed using Atlas.ti 7 [45].

#### 2.6.3 Mobile phone usage

Farmers’ current and preferred use of mobile phone was analyzed using frequency and percentage analysis.

### 2.7 Ethical statement

Prior to beginning of the study, approval was obtained from both the Laboratory of Geo- information Science and Remote Sensing—Wageningen University, The Netherlands and Bioversity international Seeds for Needs initiative scientific project leaders. Our university does not require prior ethical approval from the Social Sciences Ethics Committee for a study like this. The people who are asked to participate are not specifically vulnerable, and the interview questions were not sensitive. Oral informed consent was obtained from all respondents, who were already participating in the ongoing broader Seeds for Needs project. In Ethiopia, following the ABS proclamation 482–2006 of the Ethiopian government, farmers were interviewed after getting the necessary permission from local agricultural office administrators.

## 3. Results

### 3.1. Quantitative analysis

#### 3.1.1 Demography of farmer communities

The average age of the respondents in the three countries was similar; respondents were 47 years old on average (standard deviation (SD) = 12, range: 14–80 years) (Table 2). The majority of the respondents were male (83%). Furthermore, 90.8% of the respondents were head of the household. The respondents had different educational levels. Indian farmers in our sample were more educated than Ethiopian and Honduran farmers.

**Table 2:** Demographic characteristics of the surveyed farmers in the three countries.

Variable	India (n = 300)		Ethiopia (n = 94)		Honduras (n = 32)		
Average age ± SD	47 ± 13		48 ± 11		46 ± 14		
**Gender**	**Count**	**%**	**Count**	%	**Count**		**%**
* Male*	238	79	85	90	29		91
* Female*	62	21	9	10	3		9
**Education level**	**Count**	**%**	**Count**	%	**Count**		**%**
* Illiterate*	58	19.4	15	16.0	3		9.4
* Can read & write*	84	28.0	41	43.7	5		15.6
* Primary school*	3	1.0	24	25.5	15	47.0	
* Secondary school*	118	39.3	14	14.8	0		0
* Higher education*	34	11.3	0	0	0		0
* Missing*	3	1.0	0	0	9		28.0
**Household head**	**Count**	**%**	**Count**	%	**Count**		**%**
* Yes*	263	87.7	93	98.9	31		96.9
* No*	31	10.3	1	1.1	1		3.1
* Missing*	6	2.0	0	0	0		0

#### 3.1.2 Comparison of motivational factors within each country

**Fig 2:**
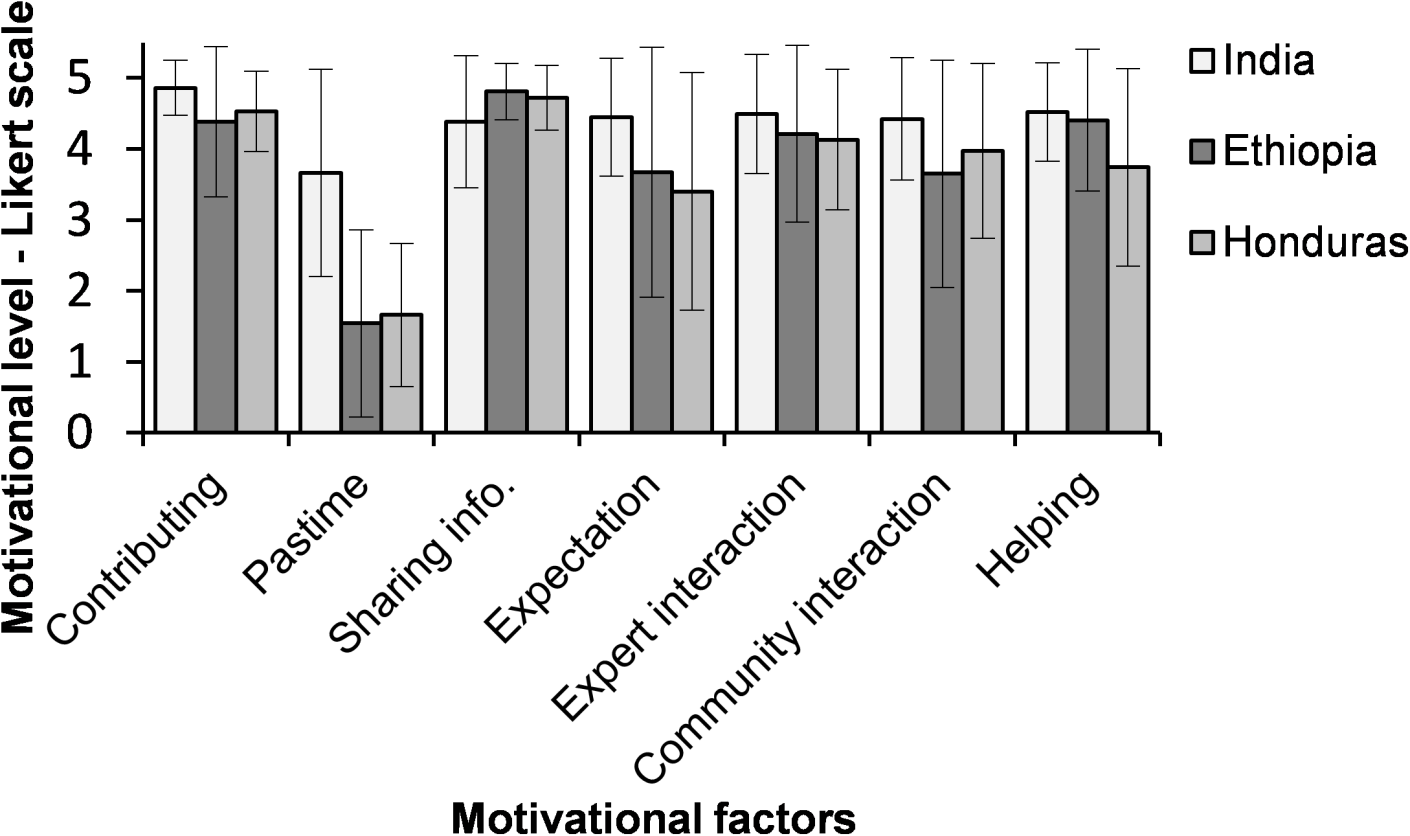
Motivational factors of farmers in the three countries (India, Ethiopia and Honduras) to participate in the crop improvement trials as citizen scientists.

Among Indian farmers, the average response rates for the motivational factors ‘*Sharing info*’, ‘*Expectation*’, ‘*Expert interaction*’, ‘*Community interaction*’ and ‘*Helping*’ were similar (average score ranging from 4.38 to 4.52) (Fig 2). However, the value of the motivational factor ‘*Helping*’ was significantly higher than ‘*Sharing info*’ (Table 3). The Indian farmers valued their participation to ‘*Contributing*’ (4.86) significantly higher and ‘*Pastime*’ (3.66) was valued significantly less compared to the other motivational factors (Fig 2 and Table 3).

**Table 3:** Differences of the motivational factors of farmers within each of the three countries’ using Friedman's test and *post-hoc* Wilcoxon signed-rank test. For codes of motivational factors see Table 1.

Comparisons	*India*				*Ethiopia*				*Honduras*			
	N	T	r	*p*-Value	N	T	r	*p*-Value	N	T	r	*p*-Value
Contributing–Pastime	282	12.23	0.45	0.000^**^	94	3331.5	0.57	0.000^**^	32	465	0.61	0.000^**^
Contributing–Sharing	281	5409	0.37	0.000^**^	94	762	0.24	0.001^**^	32	58.5	0.22	0.083
Contributing–Expectation	276	3917.5	0.02	0.000^**^	94	1022.5	0.22	0.002^**^	30	220.5	0.40	0.002^**^
Contributing–Expert interaction	284	3424	0.31	0.000^**^	94	455	0.09	0.206	31	92	0.25	0.049
Contributing–Community interaction	280	4471	0.35	0.000^**^	94	1205.5	0.27	0.001^**^	32	209.5	0.29	0.021
Contributing–Helping	282	4087.5	0.33	0.000^**^	94	414.5	0.01	0.950	31	116	0.32	0.011
Pastime–Sharing	279	5.39	0.34	0.000^**^	94	3734	0.62	0.000^**^	32	496	0.62	0.000^**^
Pastime–Expectation	274	8.09	0.38	0.000^**^	94	1956	0.50	0.000^**^	30	264	0.5	0.000^**^
Pastime–Expert interaction	282	8.68	0.37	0.000^**^	94	3299	0.56	0.000^**^	31	435	0.61	0.000^**^
Pastime–Community interaction	279	8.43	0.36	0.000^**^	94	2365	0.47	0.000^**^	32	431.5	0.59	0.000^**^
Pastime–Helping	281	8.85	0.40	0.000^**^	94	3705.5	0.57	0.000^**^	31	322.5	0.55	0.000^**^
Sharing–Expectation	273	1316.5	0.06	0.169	94	1004.5	0.38	0.000^**^	30	149.5	0.45	0.000^**^
Sharing–Expert interaction	281	1807	0.09	0.038	94	835	0.30	0.000^**^	31	121	0.38	0.003
Sharing–Community interaction	278	2105.5	0.04	0.402	94	1347	0.42	0.000^**^	32	167.5	0.38	0.002^**^
Sharing–Helping	279	1818.5	0.14	0.000^**^	94	625	0.25	0.001^**^	31	120	0.44	0.001^**^
Expectation–Expert interaction	276	1147	0.03	0.461	94	894	0.21	0.004	30	179	0.29	0.023
Expectation–Community interaction	273	1376.5	0.04	0.419	94	721	0.02	0.766	30	124	0.22	0.090
Expectation–Helping	275	903	0.07	0.086	94	1151	0.24	0.001^**^	29	132	0.2	0.125
Expert interaction–Community interaction	280	993	0.08	0.054	94	699.5	0.23	0.002^**^	31	76	0.12	0.341
Expert interaction–Helping	286	967	0.02	0.689	94	480.5	0.09	0.196	30	68	0.21	0.109
Community interaction–Helping	279	1205	0.10	0.027	94	807	0.30	0.001^**^	31	139.5	0.11	0.392

^*^: Difference within a country was statistically significant at (*P*<0.05).

^**^: Statistically significant difference detected at *P* = 0.002 (after Bonferroni adjustment for multiple comparisons).

**N**: total number of respondents, **T**: test statistics for Wilcoxon signed-rank test, **r**: effect size.

Among Ethiopian farmers, the motivational factor ‘*Sharing Info*’ was valued significantly higher and ‘*Pastime*’ was valued significantly less compared to the other motivational factors (Fig 2 and Table 3). After ‘Sharing info’, the motivations to ‘*Helping*’ and ‘*Contributing*’ were valued highest but not significantly different from ‘*Expert interaction*’ (Table 3). In addition to these two, ‘*Helping*’ and ‘*Expert interaction*’ were valued significantly higher than ‘*Community interaction*’. ‘*Expectation*’ was valued lowest after ‘*Pastime*’ and ‘*Community interaction*’, but the value was not significantly different from ‘*Expert interaction*’ and ‘*Community interaction*’.

Results for Honduras were similar to Ethiopia. Also here, ‘*Sharing info*’ was valued highest, ‘*Contributing*’ second and, ‘*Pastime*’ lowest (Fig 2 and Table 3). The average rates for the motivational factors ‘*Expert interaction*’, ‘*Community interaction*’, ‘*Helping*’ and ‘*Expectation*’ were in between and similar (average score ranging from 3.38 to 4.13) (Fig 2). The high value for ‘*Contributing*’ and low value for ‘*Pastime*’ was also found in India, while ‘*Sharing info*’ was valued less in that country.

#### 3.1.3 Comparison of motivational factors between countries

The results of the Kruskal-Wallis test show that the importance of different motivations differed between the three countries for all motivational factors (Table 4). Post-hoc pairwise comparisons using the Dunn-Bonferroni test revealed a large number of significant differences. For farmers in India ‘*Contributing*’ and ‘*Pastime*’, were more important than for farmers in Ethiopia and Honduras (Fig 2 and Table 4). Ethiopian farmers found ‘*Sharing info*’ more important for their motivation than Indian farmers, while the difference between Honduras and India was not significant. ‘*Expectation*’ was valued higher by farmers in India than farmers in Honduras and the difference with Ethiopia was almost significant. Also *‘Expert Interaction’* was more motivating for Indian farmers than for Honduran farmers, and ‘*Community Interaction*’ was more motivating in India than in Ethiopia. Extrinsic egoistic motivations (Table 1) were thus more important in India than in Honduras and Ethiopia. Lastly, ‘*Helping*’ was more motivating to Indian and Ethiopian farmers than to Honduran farmers.

**Table 4:** Comparisons of motivational factors of farmers between the three countries using the Kruskal-Wallis test followed by pairwise comparisons using the Dunn-Bonferroni test.

Motivational factor	N	Kruskal-Wallis test			Pairwise comparisons using Dunn-Bonferroni test		
		H	*df*	*p*-Value		*p*-Value	r
Contributing	410	39.15	2	0.000*	India–Ethiopia	0.000**	-0.28
					Ethiopia–Honduras	1.000	0.04
					India—Honduras	0.000**	-0.22
Pastime	408	152.47	2	0.000*	India–Ethiopia	0.000**	-0.58
					Ethiopia–Honduras	1.000	-0.03
					India—Honduras	0.000**	-0.38
Sharing info	407	18.09	2	0.000*	India–Ethiopia	0.000**	0.21
					Ethiopia–Honduras	1.000	0.07
					India—Honduras	0.259	0.09
Expectation	400	14.68	2	0.001*	India–Ethiopia	0.059	-0.12
					Ethiopia–Honduras	0.236	0.16
					India—Honduras	0.002**	-0.19
Expert interaction	409	8.56	2	0.014*	India–Ethiopia	0.561	-0.07
					Ethiopia–Honduras	0.220	0.16
					India—Honduras	0.016**	-0.16
Community interaction	406	18.303	2	0.000*	India–Ethiopia	0.000**	-0.21
					Ethiopia–Honduras	1.000	-0.03
					India—Honduras	0.081	-0.13
Helping	407	12.92	2	0.002*	India–Ethiopia	1.000	-0.01
					Ethiopia–Honduras	0.004**	0.28
					India—Honduras	0.001**	-0.20

*: Difference between the three countries was statistically significant at (P = 0.05).

**: Statistically significant difference detected at p = 0.017 (after Bonferroni adjustment for multiple comparisons).

**N**: total number of respondents, **H**: test statistics for Kruskal-Wallis test, ***df***: degree of freedom, **r**: effect size.

#### 3.1.4 Relationships among motivational factors

**Fig 3:**
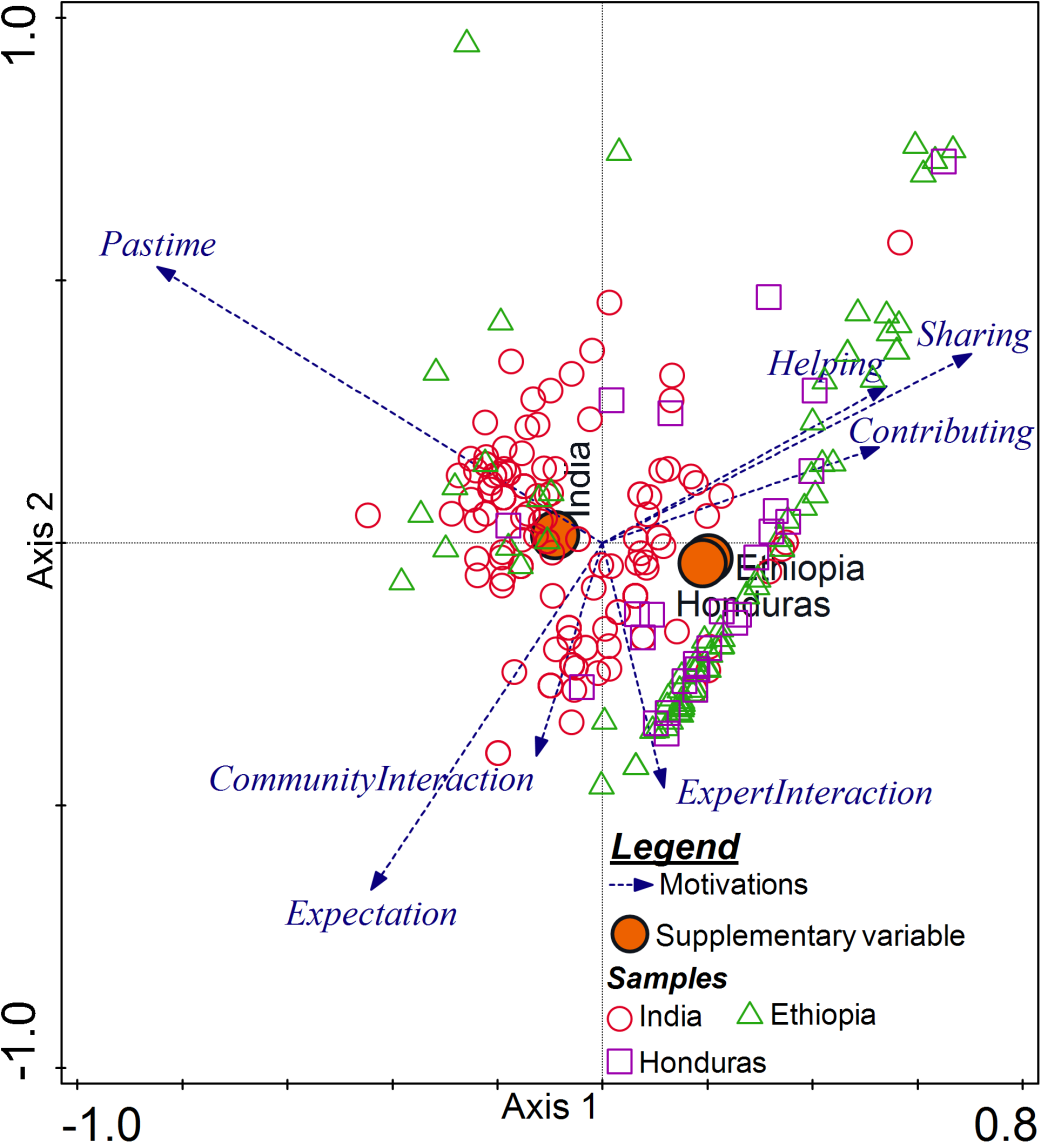
Triplot diagram showing the result of the PCA analysis of motivational factors using country as a supplementary variable (filled circle) together with samples from the three countries. The blue (dashed) vectors represent motivational factors. The circles (unfilled), triangles and squares represent samples from India, Ethiopia and Honduras, respectively.

The result of the PCA of motivational factors revealed four components. The four components explained 81.7% of the variance (S2A Appendix). The first two main components explained most of the variance (56.5%) in the motivational factors. The first component accounted for 35% of the variance and comprised of four factors (‘*Sharing info*’, ‘*Helping*’, ‘*Contributing*’ and ‘*Pastime*’ (Fig 3). While ‘*Sharing info*’, ‘*Helping*’, and ‘*Contributing*’ contribute positively to this component, ‘*Pastime*’ was negatively related. The second component accounted for 21.5% of the variance and mainly associated with ‘*Expectation*’, ‘*Expert interaction*’ and ‘*Community interaction*’ and all were negatively related to the second component. The first component also reflects what was observed earlier: the generally high importance of ‘*Sharing info*’, ‘*Helping*’ and ‘*Contributing*’ and lower importance of ‘*Pastime*’.

The vectors of the motivational factors ‘*Sharing info*’, ‘*Helping*’ and ‘*Contributing*’ point in the same direction, indicating a strong positive correlation between these three motivational factors (Fig 3). The correlation was specifically strong between ‘*Sharing info*’ and ‘*Helping*’. This implies that farmers who were motivated to share information were also motivated to help researchers and contribute to scientific research and vice versa. On the other hand, the negative relation between the factors ‘*Sharing info*’, ‘*Helping*’ and ‘*Contributing*’ and ‘*Pastime*’ vectors, suggests that farmers who were motivated either to contribute to scientific research, to help researchers or had an interest in sharing information did not consider their participation as a pastime activity. The vectors of the motivational factors ‘*Expectation*’, ‘*Expert interaction*’ and ‘*Community interaction*’ points in the same direction, suggesting a strong positive correlation among these three motivational factors (Fig 3). Some vectors (‘*Pastime*’ and ‘*Expectation*’; ‘*Contributing*’ and ‘*Expert interaction*’) cross nearly at right angle, suggesting a near zero correlation. The centroids of the supplementary variable (country) were closer for Ethiopia and Honduras than for India. This indicates that there was more similarity between Ethiopia and Honduras compared to India.

### 3.1.5 Relationship between motivational factors and farmers’ characteristics

The relationship between each of the motivational factors and farmers’ characteristics was examined using Generalized Linear Models (GLM). For educated farmers (Regr B = 0.009; *p* = 0.000), contributing to scientific research was a more important factor to participate as citizen scientists compared to less educated farmers (Table 5). Women (Regr B = 0.020; *p* = 0.004) and less educated farmers (Regr B = -0.008; *p* = 0.000) valued their participation as ‘*Pastime*’ activity more than men and educated farmers. The relationship between gender and the motivational factor ‘*Sharing info*’ was almost significant (Regr B = -0.010; *p* = 0.068). This suggests that female farmers were less interested in sharing information compared to men farmers.

**Table 5:** Generalized linear model showing relationship between motivational factors and farmers’ characteristics using pooled data from the three countries.

Motivational factors	Gender (Male, Female)		Age (in years)		Education level		Household Head (Yes, No)	
	*Regr B*^*a*^	*P-Value*	*Regr B*	*P-Value*	*Regr B*	*P-Value*	*Regr B*	*P-Value*
Contributing	0.006	0.135	0.000	0.172	0.009	0.000*	0.007	0.247
Pastime	0.020	0.004*	0.000	0.438	-0.008	0.000*	0.011	0.283
Sharing Info	-0.010	0.068	-0.000	0.717	-0.003	0.115	-0.004	0.637
Expectation	0.008	0.159	0.000	0.965	0.001	0.627	-0.005	0.511
Expert Interaction	-0.005	0.231	0.000	0.344	0.002	0.218	-0.003	0.613
Community Interaction	-0.004	0.458	0.000	0.297	0.001	0.428	-0.006	0.409
Helping	-0.007	0.107	0.000	0.156	0.001	0.596	-0.008	0.221

^a^ Regression Coefficient B

* significant at 0.05 significance level.

**Fig 4:**
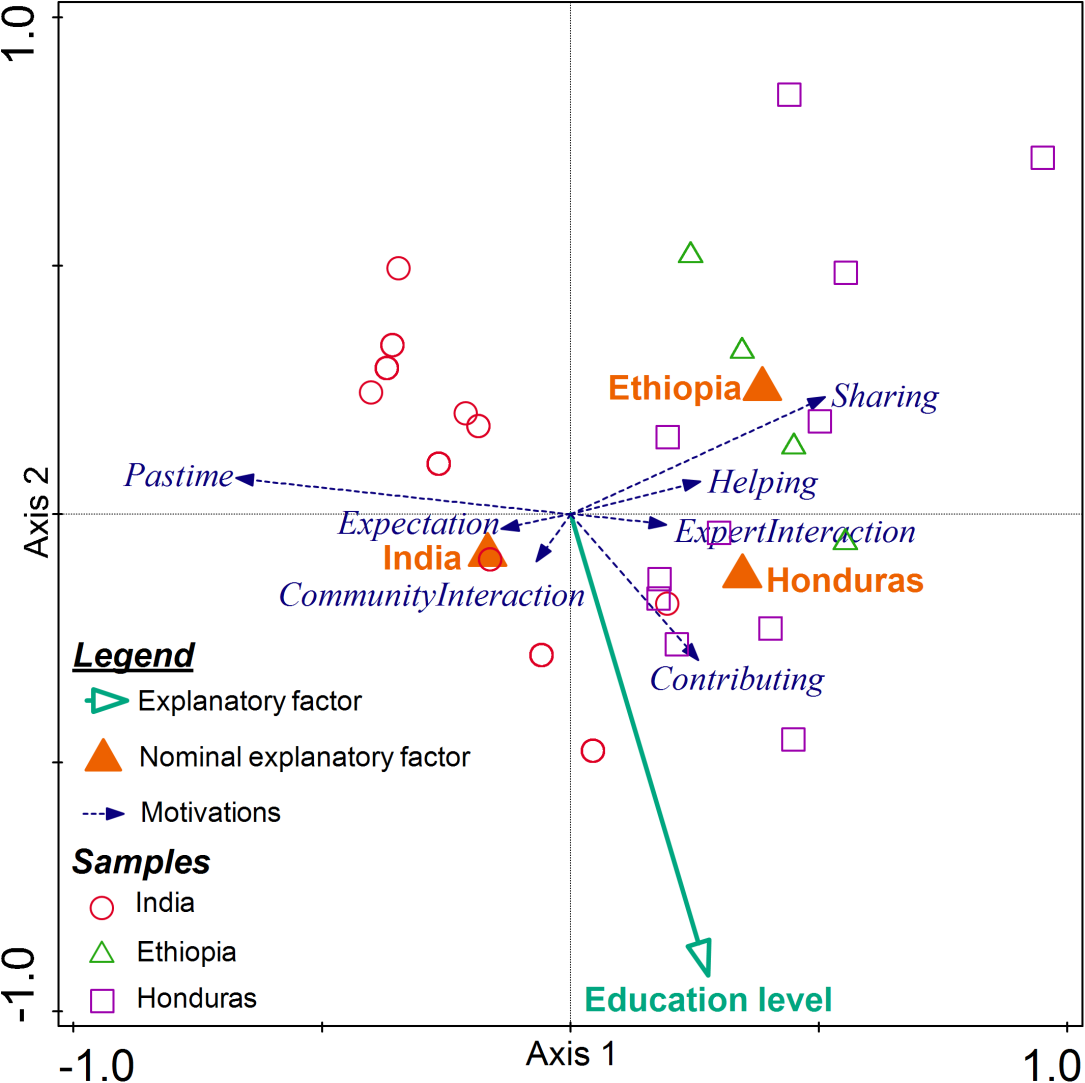
Triplot diagram showing the result of the RDA analysis of motivational factors and farmers’ characteristics together with samples from the three countries. The blue (dashed) vectors represent motivational factors, green (solid) vector denotes the explanatory factor, education level and triangles (filled) represent the nominal explanatory variable, country.

Redundancy Analysis (RDA) was used to identify the most important farmers’ characteristics that explain the variation in the set of motivational factors (Fig 4). After stepwise forward selection by RDA, only two farmers’ characteristics, country and education level, were retained (*p* < 0.05, tested by Monte Carlo permutation). The factors country and education level explained 15% and 5% of the variation in the motivational factors respectively. The education level vector points in the same direction as the motivational factor ‘*Contributing*’ vector indicating a positive correlation between education level and farmers motivation to contribute to scientific research (Fig 4). On the other hand, the vector of education level points roughly in an opposite direction to the motivational factor ‘*Pastime*’ vector. This indicates that there was a negative correlation between education level and the motivational factor ‘*Pastime*’. The vectors of the explanatory factor education level and the motivational factor ‘*Sharing info*’ cross nearly at right angle, suggesting a near zero correlation. These results also confirm what was observed earlier in the GLM analysis results (Table 5).

### 3.2 Qualitative analysis

#### 3.2.1 What do farmers expect in return from the citizen science process (‘*Expectation*’)?

**Fig 5:**
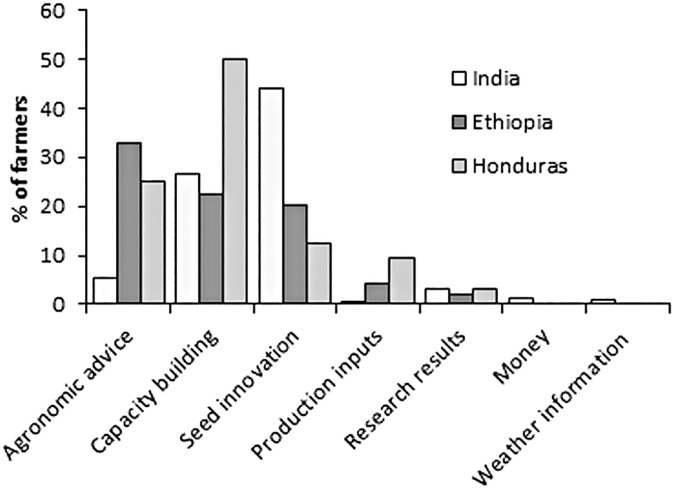
Factors which farmers expected from the citizen science process in return for their participation.

We asked farmers who had responded “Important” or “Very important” to the motivational factor ‘*Expectation*’ to specify their expectations. The main returns which farmers expect to receive from the citizen science process for participation in the crop improvement trials were: agronomic advice (e.g., weed management), capacity building (e.g., training) and seed innovation (e.g., improved seed) (Fig 5). In Ethiopia, 33% of the farmers expected to receive agronomic advice. For farmers in Honduras, capacity building was the most important factor (50%), while in India, seed innovation was what farmers (44%) expected to receive. Production inputs (e.g., fertilizers) were expected by 9.4% of farmers in Honduras. Only in India, a few farmers mentioned that they would like to receive money (1%) and weather information (1%) in return. Around 3% of the farmers in India and Honduras and around 2% of the farmers in Ethiopia indicated that they expected to receive research results from the trials they participated in.

#### 3.2.2 Additional motivations

**Fig 6:**
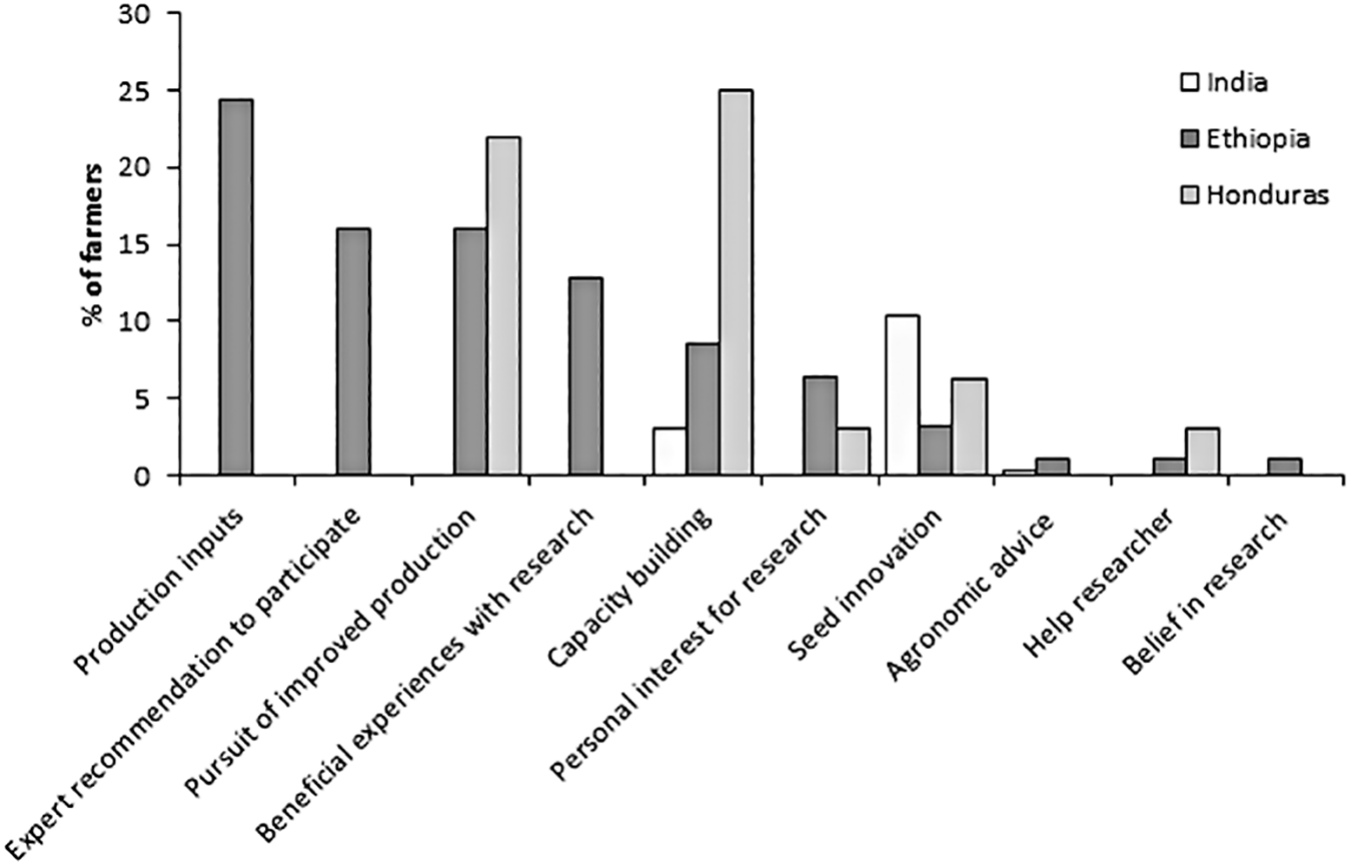
Additional motivations to participate in crop improvement trials using the crowdsourcing approach.

Farmers were also asked for any additional motivations than the pre-defined motivations (Table 1) using an open-ended question. In Ethiopia, 24.5% of the farmers mentioned production inputs (e.g., receive pesticides) as one of their motivations to participate in the crop improvement trials (Fig 6). Moreover, both expert recommendation and the desire for improved production were mentioned by 16% of the farmers. Beneficial previous experiences from research and capacity building were mentioned by 12.8% and 8.5% of the Ethiopian farmers respectively. The desire for improved production (21.9%) and capacity building (25%) were the two most mentioned motivations for farmers in Honduras. Seed innovation was the most mentioned motivation by Indian farmers (10%). Around 1% of Ethiopian and 3% of Honduran farmers had the desire to help the researcher to accomplish his/her task.

#### 3.2.3 What reward farmers expect for sharing agronomic information?

**Fig 7:**
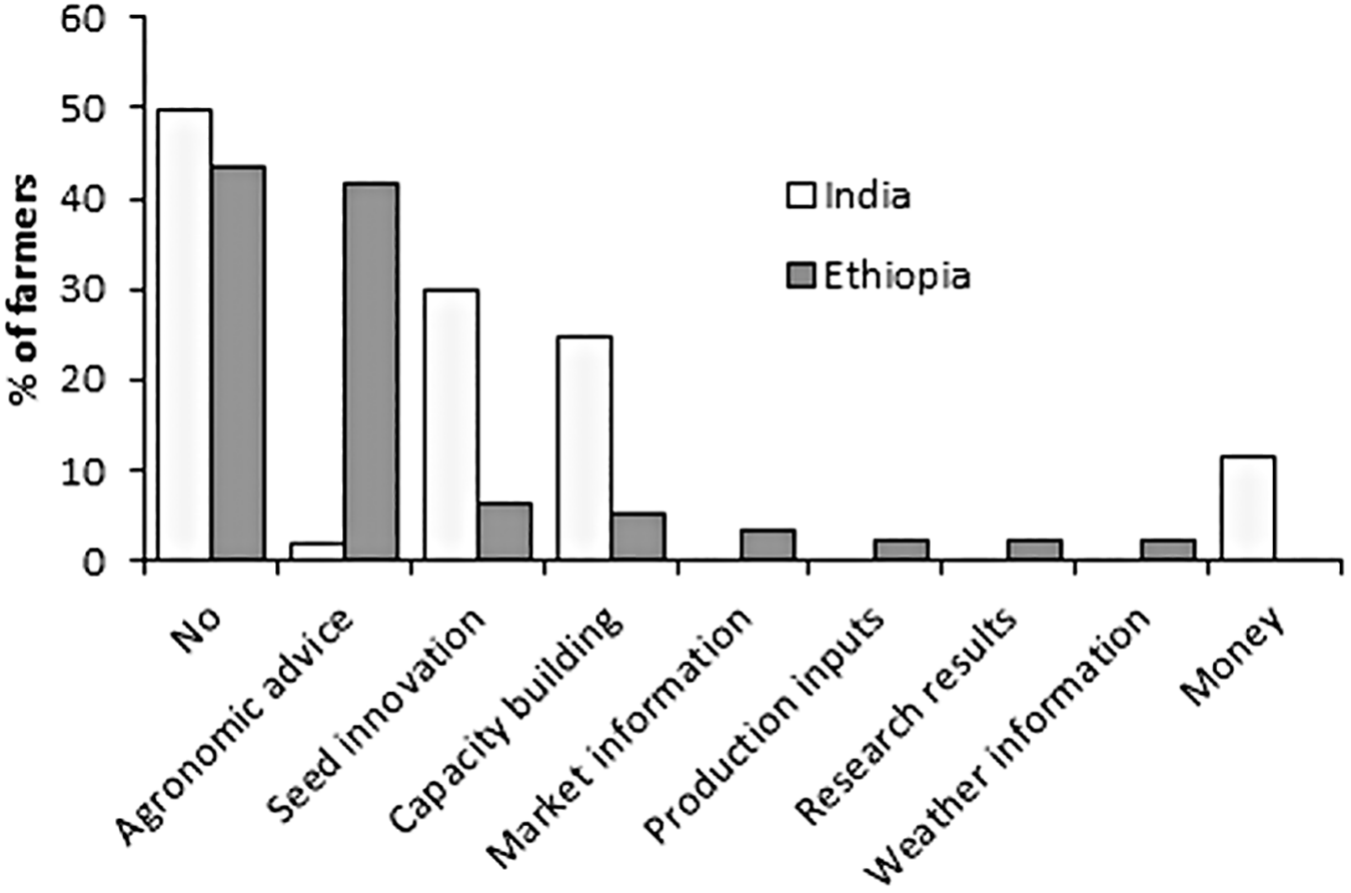
Types of rewards farmers would like to receive for sharing agronomic information.

The types of reward farmers would like to receive for sharing their agronomic information in the future was also identified using an open-ended question. Around 50% of the farmers in India and 44% in Ethiopia indicated that they do not expect any reward for sharing their agronomic information (Fig 7). However, around 42% of the farmers in Ethiopia indicated that they would like to receive agronomic information in return as a kind of reward. In India, the farmers that did indicate they expect reward, expected either seed innovation (30%), capacity building (25%) or money (11%). Information related to market and weather were also mentioned by few farmers in Ethiopia.

### 3.3 Current and preferred use of mobile phones by farmers

More than 90% of the farmers in the three countries had mobile phones (Table 6). Around 59% in India, 52% in Ethiopia and 43% in Honduras “always maintain airtime” on their mobile phones. Making and receiving calls were the two most often used functions of the mobile phone in the three countries. Using the mobile phone to browse the internet was very low in Ethiopia (1%) and Honduras (4%), but also low in India (8%). A majority of the farmers in all the three countries preferred calls over short message service (SMS) as a medium for communication. Farmers in Ethiopia used their mobile phones to access weather and market information more than Honduran and Indian farmers. Ethiopian farmers mainly used the calling feature of the phone to get market information from non-formal information channels (e.g., local traders, brokers and friends). Farmers in Ethiopia (77%) also mentioned that they used their mobile phones to receive agricultural advice. The mobile phone was mainly used by the Ethiopian farmers to communicate with extension workers and receive information (e.g., availability of inputs) and also to get extension support. Farmers used their mobile phone to receive extension support in different stages of cultivation i.e., from pre-planting (e.g., land preparation advice) until harvesting (e.g., when to harvest the crop based on the weather condition).

**Table 6:** An overview of mobile phone usage variables in the three countries.

	India	Ethiopia	Honduras
Mobile phone ownership	91%	93%	93%
Average monthly airtime expenditure (in average 2014 US$)	2.79 ± 2.18	3.58 ± 2.72	4.86 ± 3.95
*Farmers who*:			
• “Always” maintain airtime	59%	52%	43%
• “Sometimes” maintain airtime	35%	46%	39%
• “Never” maintain airtime	6%	2%	18%
*Used functions of the mobile phone*			
• Making calls	99%	100%	96%
• Receiving calls	99.5%	100%	96%
• Sending SMS messages	31%	15%	30%
• Reading SMS messages	40%	20%	32%
• Taking pictures	20%	5%	36%
• Using Internet	8%	1%	4%
Use mobile phone for personal calls	96%	100%	75%
*Use mobile phone to get*:			
• Market and weather information	43%	66%	21%
• Agricultural advice	37%	77%	21%
Able to read and understand SMS message	87%	21%	85%
*Preferred information medium for future citizen science communication*:			
• SMS	1%	8%	7%
• Calls	76%	87%	68%
• No preference	23%	5%	25%

## 4 Discussion

To increase the understanding of farmers’ motivation to participate in citizen science projects, we have interviewed 426 smallholder farmers in India, Ethiopia and Honduras as part of the “Seeds for Needs” initiative.

### 4.1. Country-by-country analysis

For Indian farmers, the collectivistic (increasing the welfare of a group that one belongs to) type of motivation (i.e., ‘*Contribute to scientific research*’) was valued more important than egoistic and altruistic motivation types (Fig 2 and Table 3). This means that Indian farmers valued their contribution to scientific research more than their participation to receive something in return (egoistic extrinsic) and interest in sharing information (egoistic intrinsic). The altruistic type of motivation ‘*Helping*’ and egoistic extrinsic type of motivation ‘*Expert Interaction*’ were the second and third important factors for Indian farmers respectively.

For Ethiopian farmers, the egoistic intrinsic type of motivation (‘*Sharing info*’) was valued more than collectivistic, altruistic and egoistic extrinsic types of motivations (Fig 2 and Table 3). The altruistic type of motivation ‘*Helping*’ and collectivistic type of motivation ‘*Contributing*’ were the second and third important factors for Ethiopian farmers respectively. Like Ethiopian farmers, for Honduran farmers, the egoistic intrinsic type of motivation (‘*Sharing info*’) was valued more important than collectivistic, altruistic and egoistic extrinsic types of motivations (Fig 2 and Table 3). The collectivistic type of motivation ‘*Contributing*’ and egoistic extrinsic type of motivation ‘*Expert Interaction*’ were the second and third important factors for Honduran farmers respectively. The difference in motivations of farmers between the three countries suggests that future citizen science projects targeting the farming community in developing countries might need to consider different approaches to attract and retain farmer citizen scientists. Factors that motivate farmers in a specific country might not necessarily motivate farmers in another country.

### 4.2 Comparisons between countries

Comparison of motivational factors between the three countries revealed that Indian farmers valued their contribution to scientific research (‘*Contributing*’) more than Ethiopian and Honduran farmers, making ‘*Contributing*’ a more salient motivator for Indian farmers (Fig 2 and Table 4). This might be because our sampled farmers in India are more educated (Table 2) and hence, have better understanding and perception of their participation to contribute to scientific research. Participation to ‘*Contributing*’ was still the second and third most important motivational factor for Honduran and Ethiopian farmers, respectively. This indicates that the motivational factor ‘*Contributing*’ is in general an important factor for farmers in all the three countries to participate as citizen scientists.

For Ethiopian and Honduran farmers, ‘*Interest in Sharing information*’ was the first ranked motivational factor. By sharing their (mostly agronomic) information, farmers thought that they would receive expert advice on how to improve their crop production. As revealed from the open-ended questions (Fig 5), 33% of Ethiopian and 25% of Honduran farmers would like to receive agronomic advice for their participation as citizen scientists’. In order to achieve this need of farmers, sharing their agronomic information with experts or researchers is necessary. Besides, for experts to deliver helpful agronomic advice for the farmers, receiving information from farmers about the different agronomic practices performed in the farmers’ fields and socio-economic conditions of the farmers helps to provide a set of site-specific agronomic advices [46–49]. In the context of variety selection, receiving information from farmers about their variety preferences can be used by agro-dealers and provide preferred seed varieties to farmers in the following cropping season.

For both Ethiopian and Indian farmers, the motivational factor ‘*Helping*’ was the second ranked motivational factor. The direct reason or ultimate goal for this can be because farmers in Ethiopia and India have the desire to help researchers or experts to accomplish their tasks. However, these farmers also might have thought, if they help the researchers or experts to get their job done, researchers or experts in return will help them when they have problems (e.g., visiting an agronomic expert when a farmer has an urgent question). According to Batson et al. [31], a goal can be either ultimate or instrumental. An ultimate goal is the valued state the individual is seeking to reach, while the instrumental goals are sought as they act as stepping stones to one’s ultimate goals. In this situation, farmers might use the opportunity of ‘*Helping*’ as a stepping stone to their ultimate goals. In this case, the ultimate goal of farmers is to produce better yield and for this they need expert advice for the different problems they might face over the growing season.

### 4.3 Generalization and reliability of results

The study was exploratory and, as such does not claim to statistically represent farmers in all the three countries (e.g., in terms of age and gender). However, the findings give insights on motivations that are likely to be common among smallholder farmers in Ethiopia, India and Honduras.

An earlier exploratory study, by Johnson et al. [37], assessed the motivation of citizen science volunteers in India to participate in wildlife conservation projects, and one of the motivations to participate was ‘concern to the environment and wildlife conservation’. This motivation can be categorized under the theme “collectivistic motivation” [31], which was also important for Indian farmers in our study. The study of Rotman et al. [50] showed the importance of ‘personal interests’ for volunteers to participate in citizen science projects in Costa Rica. In our study, Honduran farmers valued their participation to ‘share information’ mainly with experts and would like to receive feedback in return (egoistic). Getting similar results both from volunteer citizen scientists in Costa Rica and Honduras highlights that there is some sort of similarity for people to participate in citizen science in central America. In general, the more studies that will be performed, in the more regions, the more can be said about generalization of results.

We took several precautions to ensure good data quality and responsiveness of the farmers. These included adhering a similar approach in the three countries where the study was conducted, having the interviewer being assisted by local people to make the farmers comfortable, and using a well-developed methodology used by previous studies (Table 1). In order to cross-check the answers provided by the farmers, the use of role-playing games might be used in future studies. Letting the farmers play games, designed to capture the motivation of a farmer to participate in citizen science, might be used to triangulate what has been said during the interview. For example, the study of Villamor et al. [51] used role-playing games to identify gender-specific preferences for annual crops and tree-based agroforestry systems, and the underlying motivation of those preferences.

### 4.4 Comparison with other citizen science applications

The nature of the Seeds for Needs initiative is different from most other citizen science projects in that it works with smallholder farmers in developing countries. Therefore, we discuss if the findings of other studies on the role of motivation in citizen science can be generalized to this type of citizen science.

The finding that citizen scientists in Seeds for Needs have a high motivation to contribute to science (‘*Contributing*’) is in line with many other studies of citizen science projects in applications related to astronomy [16, 33], in understanding the three-dimensional structures of protein (Foldit, example of citizen science game [23]), in measuring aerosols using smartphones (e.g., iSPEX; [24]), in health [52, 53] and in collaborative distributed computing projects [34]. In ecology-based citizen science projects, Rotmal et al. [25] found that egoism (one’s own welfare) was the most important motivational factor during initial participation. We also found the same result (egoistic intrinsic i.e., ‘*Sharing Info*’) for both Ethiopian and Honduran farmers. However, Indian farmers had a more collectivistic motivation (‘*Contributing*’) at the start of their participation.

Although reasons such as enjoyment of the activity (‘*Pastime*’) can be an important reason to participate in other citizen science projects [23, 36], it was not particularly an important motivational factor for farmers in the three countries who participated in the “Seeds for Needs” initiative. Possibly, the close relation of the project with the professional activities of the participants might have created the difference here. Unlike other citizen science projects that include going outdoors to explore and record observational data in nature (e.g., bird watching; [54]), for the smallholder farmers, testing the different seed varieties on their farming condition is crucial for their livelihood. Seed is an important production input for farmers and they would like to participate and perform variety selection as part of their main task, not as a ‘*Pastime*’ activity. However, there is a variation between the farmers in the three countries on how they perceive the motivational factor ‘*Pastime*’. Indian farmers significantly scored higher than Ethiopian and Honduran farmers on the motivational factor ‘*Pastime*’ (Fig 2 and Table 4). This indicates that there are still some farmers who enjoyed their participation as citizen scientists more than others and these farmers might be important agents to promote citizen science locally in the future. The study of Johnson et al. [37] discussed that when citizens are interested in one or more environmental issues, they seek out citizen science opportunities to gain expertise through participation and diffuse acquired skills and knowledge to peers through social networks, education of other non-scientist citizens.

The lower scoring of the motivational factor ‘*Pastime*’ by the farmers might also be that farmers may have interpreted this as being about “funny fun”, and not about “serious fun” (e.g., the difference between card playing and enjoying our job). Even though farmers scored low for ‘*Pastime*’ compared to the other motivational factors, this does not mean that enjoyment should not be an important ingredient in designing a future digital citizen science system. It could also be that the citizen science in this project was ‘not fun enough’.

In all the three countries, the motivational factor ‘C*ommunity interaction*’ was valued less by farmers for their participation (Fig 2 and Table 3). This might be because farmers mostly test the different varieties in their own fields and farmers did not have much opportunity to interact with other farmers during the variety selection process. However, social interaction was an important motivational factor for other citizen science projects [33, 34]. This could also point to the fact that farmers in our study areas conceive their productive activities as centered on the household and not the community, which may explain the difficulty of community-based approaches to participation in agricultural projects (e.g., [55]). Also, motivational factors may change over time [25]. It remains to be seen if citizen science projects have the potential of strengthening local communities around agricultural experiments.

### 4.5 Grouping of farmers based on motivations

The result of the PCA showed that motivational factors in the same category i.e., from egoistic extrinsic type such as ‘*Expectation*’, ‘*Expert interaction*’ and ‘*Community interaction*’ were positively correlated (Fig 3). The strong correlation between these extrinsic motivations indicates that there was a group of farmers who were motivated extrinsically. On the other hand, the positive strong correlation among the motivational factors from different motivation types ‘*Sharing info*’ (egoistic intrinsic), ‘*Helping*’ (altruistic) and ‘*Contributing*’ (collectivistic) indicate another group of farmers who participated because they had different types of motivations. This indicates that many farmers did not have only one type of motivation to participate; rather they had different types of motivations. Similar results i.e., people having different types of motivations to participate in citizen science were also found in other studies [16, 23]. Motivational factors, ‘*Pastime*’ and ‘*Sharing info* ‘ both egoistic intrinsic, were oppositely valued by the farmers. This indicates that farmers’ motivations need to be assessed as specific as possible rather than generalize it under major motivation types. If the citizen science approach had to tailor to different groups of farmers with different motivations, these two groupings of farmers can be used to divide the farmers into major groups that can be handled differently.

### 4.6 Effects of farmers’ characteristics on farmers’ motivations

The relationship between motivational factors and farmers’ characteristics using redundancy analysis revealed that country is a main factor explaining the variation in the motivations of the farmers (Fig 4). This might be because of cultural differences between the three countries. An in-depth comparison of cultures is beyond the scope of this paper. However, for example according to the Hofstede's cultural dimensions, Ethiopian and Honduran nationals are on average more collectivistic compared to Indian nationals ([56, 57]; https://geert-hofstede.com/countries.htmlhttps://geert-hofstede.com/). This might be the reason why farmers in Ethiopia and Honduras valued sharing information more than Indian farmers.

Farmer’s characteristics, country and education level explained only 20% of the total variation in the motivational factors (S2C Appendix). The large unexplained variation indicates that there were other factors which were not considered in the current study but might had been relevant to explain the variation in the motivations of farmers in the three countries.

### 4.7 Prospects of gamification

The findings about motivational factors in farmers’ participation shed light on the prospects of gamification in this type of citizen science and which motivational factors it needs to support. Even though there are important differences between groups of participants with different educational level and gender, at the same time the diversity of motivations within each group imply that gamification should tailor to a number of different motivations at the same time to be inclusive. There is a low emphasis on ‘*Pastime’* as a motivation for participating in tricot citizen science trials and it has a negative correlation with other motivational factors of more weight. This finding suggests that a careful approach is needed to support the enjoyment of tricot trials. Even though game-like elements in a broad sense may play a role, it will be important to determine if each element is appropriate in this context. In any case, gamification through the unreflective adoption of game elements that emphasize extrinsic motivation (scoreboards, badges, etc.) will likely be counterproductive. Intrinsic motivation features highly in the motivation factors that participating farmers score highly in all three study areas. Likewise, Deterding [18] emphasizes intrinsic motivation for enjoyable game experiences, with references to Self-Determination Theory. According to this theory, intrinsically motivating activities are those that the individual finds interesting and performs without any kind of conditioning, just by the mere pleasure of carrying them out, supported by autonomy (which requires the task to be voluntary), the need to feel competent and efficient and to feel connected to other persons [15, 58]. The relatively high scores for intrinsic motivation factors from the current study reinforces this view.

### 4.8 Future potential of mobile phone as technological interface for citizen science

Volunteers’ participation in digital citizen science activities is grounded on two facilitating pillars: a motivational, and a technological pillar [26]. The results of this study show the high initial motivation of smallholder farmers to act as citizen scientist. The next important issue is to assess the technological pillar for digital citizen science. For smallholder citizen scientist farmers, mobile phone is the most accessible technology to use and provide their experimental results. Interestingly, an overwhelming majority of the sampled farmers in the present study have mobile phones (Table 6). This result shows a promising potential as most of the farmers have the mobile telephone technology to provide their experimental results. In terms of preferences, farmers preferred calling over SMS because of their illiteracy. This means citizen science projects targeting the farmers’ community need to consider to include features like Interactive Voice Response (IVR) systems as a data collection mechanism to harness the full potential of mobile phone as a citizen science data collection tool. Recent examples of using IVR to collect food security indicators at the household level shows its huge potential to be used in citizen science projects which target the farming community [59]. Furthermore, mobile phones also have the potential to facilitate the interaction between the farmers and experts. In earlier examples, in the Digital Early Warning Network (DEWN) project, an initiative at the International Institute of Tropical Agriculture (IITA), farmers send text messages to researchers about incidence of Cassava Mosaic Disease (CMD) and Cassava Brown Streak Disease (CBSD) and receive disease control options in return [60]. Having detailed agronomic data from farmers participating as citizen scientists can also be used by researchers to identify the key causes of the yield gap, in order to prioritize efforts in research and extension [61]. Moreover, participating in citizen science projects and share their information using mobile phones (e.g., land information) can even give farmers the opportunity to get connected across the globe and learn on how to manage their plot of land from other farmers with similar land characteristics (www.landpotential.org/; [62]).

## 5 Conclusions

This study explored the motivations of farmers to participate as citizen scientists in crop improvement trials in three countries: Ethiopia, Honduras and India. The most pronounced motivation for Indian farmers was the desire to contribute to scientific research (i.e., collectivistic). For Ethiopian and Honduran farmers, the motivation ‘*Interest in sharing information*’ (i.e., egoistic intrinsic) was more salient than the other types of motivations. ‘*Pastime*’ was in general less motivating compared to the other motivational factors. Two major groups of farmers could be distinguished for future design: one motivated by sharing information (egoistic intrinsic), helping (altruism) and contribute to scientific research (collectivistic) and one motivated by egoistic extrinsic factors (expectation, expert interaction and community interaction). Around half of the farmers expected something in return from the citizen science process. Agronomic advice, capacity building and seed innovation were the most needed incentives.

The majority of the farmers have mobile phones and they are already using their mobile phones to access extension advice and market information. Even if the farmers who participated in the present study did not use their mobile phones to provide their experimental results yet, we can conclude that there is a high potential for farmers to use their mobile phones to provide information from their experimental results. However, since there are many farmers who are not educated, it is recommended to introduce easy to use mechanisms (e.g., Interactive Voice Response).

We conclude that motivations to participate in citizen science are different for smallholders in agriculture compared to other sectors. Citizen science does have high potential, but easy to use mechanisms are needed. Moreover, gamification may increase the egoistic intrinsic motivation of farmers.

## Supporting information

S1 Appendix Motivation interview questions. (DOCX)

S2 Appendix Summary of PCA and RDA results. (DOCX)

S3 Appendix GLM result. (DOCX)
