# Supplementary material for: What are the prospects for citizen science in agriculture? Evidence from three continents on motivation and mobile telephone use of resource-poor farmers
Source: PLoS One. 2017 May 4;12(5):e0175700. doi: 10.1371/journal.pone.0175700 (PMC5418078; doi:10.1371/journal.pone.0175700)
Supplement: S1 Appendix — (DOCX) [file pone.0175700.s001.docx]

**S1 Appendix: Motivation interview questions**

The main purpose of this survey is to assess the motivations of farmers for participating in Crowdsourcing crop improvement trials and to assess farmer’s usage of mobile phones.

Name of interviewer: ____________________________ Country: ____________________________

1. **Background information of the respondent**

Date of interview: ____________________________ Village name: ________________________

Full name of respondent: _________________________________________________________

Is the respondent head of the household: Yes [ ] No [ ] Age of respondent (Years): _________

Gender: Male [ ] Female [ ] Education level of the respondent: ____________________________

1. **Electricity**

In your community, how often you don’t have electricity**?**

[ ] Always there is electricity

[ ] Once a week

[ ] 1 hr. a day

[ ] 6 hrs. a day

[ ] 12 hrs. a day

[ ] Other quantity hours: _____________________________

[ ] No electricity

1. **Use of mobile phones**

| Question | Answer |
| --- | --- |
| 1. Do you have a mobile phone? | No [ ] Yes [ ] |
| 1. If yes, who owns the mobile phone? | [ ] Farmer /respondent  [ ] Other family member (e.g. son) |
| 1. Do you usually have money/air time in the mobile phone? | [ ] Always  [ ] Sometimes  [ ] Almost never  [ ] Never |
| 1. How much money do you spend for local call per month (in local currency)? |  |
| 1. Which functions of the mobile phone are you using? | [ ] Making calls  [ ] Receiving calls  [ ] Send messages  [ ] Read messages  [ ] Taking pictures  [ ] Internet  [ ] None of the above  [ ] Other(s): |
| 1. How frequently do you use these functions? | Make calls:  Receive calls:  Send Messages:  Read messages:  Take picture:  Internet: |
| 1. Do you use the mobile phone for personal calls? | No [ ] Yes [ ] |
| 1. Do you use the mobile phone for getting information about markets, weather, agricultural advice, etc.? | No [ ] Yes [ ] |
| 8.1. If yes, please explain for which specific purpose you use the mobile phone |  |
| 1. Did you send message via SMS? | No [ ] Yes [ ] |
| 1. Did you receive message via SMS? | No [ ] Yes [ ] |
| 1. If Yes for Q. 10, could you read and understand the message? | No [ ] Yes [ ] |
| 1. If No for Q. 10, why not? Please explain | |
| 1. Do you prefer to receive messages or receive calls? | [ ] Calls  [ ] Messages  [ ] No preference |
| 1. Can you explain why you prefer messages or calls? | |

1. **Motivation**

| 1. Are you willing to participate in the trials and share information? | [ ] Yes [ ] No |
| --- | --- |
| 1.1 If Yes or No, why? | |
| 1.2 (In the case of “Yes, but only with conditions”)  What are your conditions? | |
| 1. If your answer for Q1 is “Yes”, what is your motivation to participate? Please express what motivates you by checking one box for each question (questions 2.1 until 2.8) | |

**I participate in the trials and provide information :-**

| 2.1 | To contribute to scientific research: | *Very important* | *Important* | | *Neutral* | | | *Not important* | | *Not important at all* |
| --- | --- | --- | --- | --- | --- | --- | --- | --- | --- | --- |
| 2.2 | To pass my free time (hobby): | *Very important* | *Important* | | *Neutral* | | | *Not important* | | *Not important at all* |
| 2.3 | Because providing/sharing information is interesting for me: | *Very important* | *Important* | | *Neutral* | | | *Not important* | | *Not important at all* |
| 2.4 | Because I expect something in return from the technician/researcher: | *Very important* | *Important* | | *Neutral* | | | *Not important* | | *Not important at all* |
|  | What do you expect in return? | | | | | | | | | |
| 2.5 | To interact/network with technicians/ researchers: | *Very important* | | *Important* | | *Neutral* | *Not important* | | *Not important at all* | |
| 2.6 | To interact/network with the community: | *Very important* | | *Important* | | *Neutral* | *Not important* | | *Not important at all* | |
| 2.7 | To help the technician/researcher to get his/her research done: | *Very important* | | *Important* | | *Neutral* | *Not important* | | *Not important at all* | |
| 2.8 | If you have other motivation which is not mentioned, please specify |  | | | | | | | | |

1. If you are asked to share information about your farm in the future would you expect recompense? If so, what kind of recompense?
2. Would you like to collaborate more? Yes [ ] No [ ]

4.1 In case of *“*Yes*”,* how could you collaborate more?

[ ] Give more information about my trials

[ ] Sharing my harvest seeds with others

[ ] Collaborate with others who participate

[ ] Explain the trials to others who don’t participate yet

[ ] Accompany the researcher/technician to other localities (e.g. to go to a new community and explain the trials)

[ ] Somethings else: _____________________________________________________
