# Supplementary material for: What are the prospects for citizen science in agriculture? Evidence from three continents on motivation and mobile telephone use of resource-poor farmers
Source: PLoS One. 2017 May 4;12(5):e0175700. doi: 10.1371/journal.pone.0175700 (PMC5418078; doi:10.1371/journal.pone.0175700)
Supplement: S2 Appendix — (DOCX) [file pone.0175700.s002.docx]

**S2 Appendix: Summary of PCA and RDA results**

1. Summary of PCA result

| **Statistic** | **Axis 1** | **Axis 2** | **Axis 3** | **Axis 4** |
| --- | --- | --- | --- | --- |
| Eigen values | 0.3490 | 0.2156 | 0.1533 | 0.0996 |
| Explained variation (cumulative) | 34.9 | 56.46 | 71.79 | 81.74 |

1. Summary of RDA result

| **Statistic** | **Axis 1** | **Axis 2** | **Axis 3** | **Axis 4** |
| --- | --- | --- | --- | --- |
| Eigenvalues | 0.1755 | 0.0200 | 0.0055 | 0.2251 |
| Explained variation (cumulative) | 17.55 | 19.55 | 20.10 | 42.60 |
| Pseudo-canonical correlation | 0.7188 | 0.4024 | 0.2263 | 0.0000 |
| Explained fitted variation (cumulative) | 87.31 | 97.29 | 100.00 |  |

1. Forward selection summary result

| **Name** | **Explains %** | **Contribution %** | **Pseudo-F** | **P** |
| --- | --- | --- | --- | --- |
| Country - India | 14.2 | 68.8 | 61.4 | 0.002 |
| Education level | 5.2 | 25.3 | 24.0 | 0.002 |
| Country - Ethiopia | 0.7 | 3.3 | 3.1 | 0.014 |
| Country - Honduras | 0.7 | 3.3 | 3.1 | 0.018 |
