# Supplementary material for: What are the prospects for citizen science in agriculture? Evidence from three continents on motivation and mobile telephone use of resource-poor farmers
Source: PLoS One. 2017 May 4;12(5):e0175700. doi: 10.1371/journal.pone.0175700 (PMC5418078; doi:10.1371/journal.pone.0175700)
Supplement: S3 Appendix — (DOCX) [file pone.0175700.s003.docx]

S3 Appendix: Generalized linear model showing relationship between motivational factors and farmer’s characteristics in:

1. India

| **Motivational factors** | **Gender**  **(Male, Female)** | | **Age**  **(in years)** | | **Education**  **level** | | **Household**  **Head**  **(Yes, No)** | |
| --- | --- | --- | --- | --- | --- | --- | --- | --- |
|  |  |  |  |  |  |  |  |  |
|  | *Regr B^a^* | *P-*  *Value* | *Regr B* | *P-*  *Value* | *Regr B* | *P-Value* | *Regr B* | *P-Value* |
| Contribute | 0.005 | 0.181 | 0.000 | 0.839 | **0.009** | **0.000*** | **0.010** | **0.046*** |
| Pastime | 0.000 | 0.976 | 0.000 | 0.742 | **-0.015** | **0.000*** | 0.003 | 0.681 |
| Sharing Info | -0.005 | 0.323 | 0.000 | 0.134 | 0.002 | 0.222 | 0.006 | 0.373 |
| Expectation | 0.001 | 0.790 | 0.000 | 0.389 | -0.002 | 0.264 | -0.003 | 0.664 |
| Expert Interaction | -0.001 | 0.854 | -8E-5 | 0.526 | **0.003** | **0.034*** | -0.002 | 0.741 |
| Community Interaction | -0.001 | 0.761 | -3.75E-5 | 0.820 | 0.002 | 0.236 | -0.009 | 0.150 |
| Helping | 0.000 | 0.931 | -5.87E-5 | 0.653 | 0.002 | 0.094 | -0.005 | 0.316 |

1. **Ethiopia**

| **Motivational factors** | **Gender**  **(Male, Female)** | | **Age**  **(in years)** | | **Education**  **level** | | **Household**  **Head**  **(Yes, No)** | |
| --- | --- | --- | --- | --- | --- | --- | --- | --- |
|  |  |  |  |  |  |  |  |  |
|  | *Regr B^a^* | *P-*  *Value* | *Regr B* | *P-*  *Value* | *Regr B* | *P-Value* | *Regr B* | *P-Value* |
| Contribute | 0.026 | 0.068 | **0.001** | **0.029*** | **0.020** | **0.000*** | -0.014 | 0.739 |
| Pastime | 0.027 | 0.097 | 0.000 | 0.332 | -0.006 | 0.315 | -0.024 | 0.596 |
| Sharing Info | -0.003 | 0.828 | 0.000 | 0.777 | -0.005 | 0.299 | -0.014 | 0.725 |
| Expectation | 0.002 | 0.920 | -9.63E-5 | 0.879 | -0.010 | 0.174 | 0.043 | 0.490 |
| Expert Interaction | -0.012 | 0.423 | 0.000 | 0.706 | 0.003 | 0.529 | -0.005 | 0.910 |
| Community Interaction | -0.015 | 0.425 | 0.000 | 0.667 | -0.005 | 0.421 | 0.030 | 0.575 |
| Helping | -0.025 | 0.124 | -6.94E-5 | 0.883 | 0.003 | 0.589 | -0.016 | 0.727 |

1. **Honduras**

| **Motivational factors** | **Gender**  **(Male, Female)** | | **Age**  **(in years)** | | **Education**  **level** | | **Household**  **Head**  **(Yes, No)** | |
| --- | --- | --- | --- | --- | --- | --- | --- | --- |
|  |  |  |  |  |  |  |  |  |
|  | *Regr B^a^* | *P-*  *Value* | *Regr B* | *P-*  *Value* | *Regr B* | *P-Value* | *Regr B* | *P-Value* |
| Contribute | -0.027 | 0.432 | 0.001 | 0.429 | **0.022** | **0.026*** | 0.035 | 0.514 |
| Pastime | -0.004 | 0.885 | 0.000 | 0.673 | **-0.017** | **0.043*** | **0.131** | **0.004*** |
| Sharing Info | 0.017 | 0.600 | -3.036E-5 | 0.959 | 0.016 | 0.082 | 0.018 | 0.722 |
| Expectation | 0.077 | 0.178 | 0.001 | 0.277 | 0.001 | 0.966 | -0.164 | 0.063 |
| Expert Interaction | 0.003 | 0.926 | 0.000 | 0.669 | -0.001 | 0.915 | **-0.156** | **0.003*** |
| Community Interaction | **-0.126** | **0.000*** | 0.000 | 0.455 | 0.004 | 0.671 | **0.202** | **0.000*** |
| Helping | 0.060 | 0.196 | **-0.002** | **0.016*** | -0.025 | 0.068 | -0.065 | 0.361 |
